# Enhancement of mosquito trapping efficiency by using pulse width modulated light emitting diodes

**DOI:** 10.1038/srep40074

**Published:** 2017-01-06

**Authors:** Yu-Nan Liu, Yu-Jen Liu, Yi-Chian Chen, Hsin-Yi Ma, Hsiao-Yi Lee

**Affiliations:** 1 Department of Electrical Engineering, National Kaohsiung University of Applied Sciences, Kaohsiung 80778, Taiwan; 2 Department of Industrial Engineering and Management, Minghsin University of Science and Technology, Hsinchu 30401, Taiwan; 3 Department of Graduate Institute of Clinical Medicine, Kaohsiung Medical University, Kaohsiung 807, Taiwan

## Abstract

In this study, a light-driving bug zapper is presented for well controlling the diseases brought by insects, such as mosquitoes. In order to have the device efficient to trap the insect pests in off-grid areas, pulse width modulated light emitting diodes (PWM-LED) combined with a solar power module are proposed and implemented. With specific PWM electric signals to drive the LED, it is found that no matter what the ability of catching insects or the consumed power efficiency can be enhanced thus. It is demonstrated that 40% of the UV LED consumed power and 25.9% of the total load power consumption can be saved, and the trapped mosquitoes are about 250% increased when the PWM method is applied in the bug zapper experiments.

In tropical and subtropical regions of the world, much of the local populations are at risk due to rampant mosquito-borne diseases. For instance, the Filariasis and West Nile virus are transmitted by Culex quinquefasciatus^1^, the Japanese Encephalitis virus (J.E.V.) is transmitted by Culex annulus, and Dengue Fever and Zika virus^2^,^3^ are transmitted by Aedes aegypti and/or Aedes albopictus. The Zika virus has become a recent epidemic in Central and South American countries especially.

Presently, with exclusion of the J.E.V., there are no effective vaccines that can prevent these aforementioned viruses. Epidemic prevention mainly involves the use of chemical agents for mosquito eradication. However, such a method can cause drug resistance in mosquitoes as well as environmental pollution. Browne *et al*. found that mosquitoes are highly sensitive to light intensity, direction, wavelength, color, and contrast ratio^4^,^5^. Section analysis of the mosquito eye conducted by Kay *et al*. revealed its sensitivity to ultraviolet (UV) rays^6^. Shimoda *et al*. used LED light sources with low energy consumption and specific wavelengths to pest control^7^. Field and laboratory investigations into mosquito response to artificial light have shown, that blue and green light is often more attractive than that in the yellow-orange and red regions of the visible spectrum^8^. Thus, the effectiveness of luminous traps for vectoring mosquitoes has been supported by empirical evidence and they have come into mass production and wide use.

The majority of modern luminous bug zappers use >10 W fluorescent lamps (FLs) as light sources. FLs are inexpensive, yet their UV rays are not efficient in terms of attracting mosquitoes. Under the same UV output, light-emitting diodes (LED) can save over 30% more electric power than FLs^9^. Moreover, when used outdoors without utility power supply, FL bug zappers fed only by battery do not last long due to high power consumption. In order to reduce power consumption, Ayan Kumar *et al*.^10^ used a sensor that determined initiation of the capture device. Therefore, current research on bug zappers should focus on improvements in both zapper efficiency and the ability to operate outdoors.

Due to short response time, LED output can be pulse width modulated (PWM) via high-frequency electrical circuits, The PWM is an energy-saving technology, and we try to use the intermittent PWM signals to increase the insect attraction to light. This study proposes an intelligent light-driven bug zapper integrating a 3 W UV LED module and a pulse signal circuit to attract mosquitoes. A solar power generation system is used to provide power for the bug zapper, which solved the issue of non-availability of utility power outdoors. Our experimental results will show that the use of a PMW-LED module can enhance the bug zapper’s capability to capture insects meanwhile increasing power efficiency by reducing power consumption from 1.5 W to 0.9 W, which can provide better mosquito eradication and improved power efficiency.

## Experimental Results

**Figure 1:**
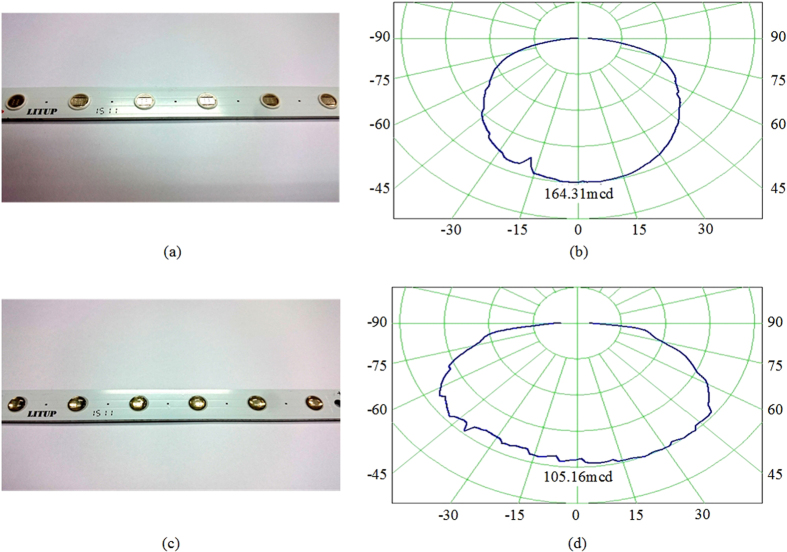
(**a**) The module of UV LED light without lens. (**b**) The 2D luminous intensity distribution curves of the module of UV LED without lens (Unit: mcd/Klm). (**c**) The module of UV LED light with lens. (**d**) The 2D luminous intensity distribution curves of the module of UV LED with lens (Unit: mcd/Klm).

**Figure 2:**
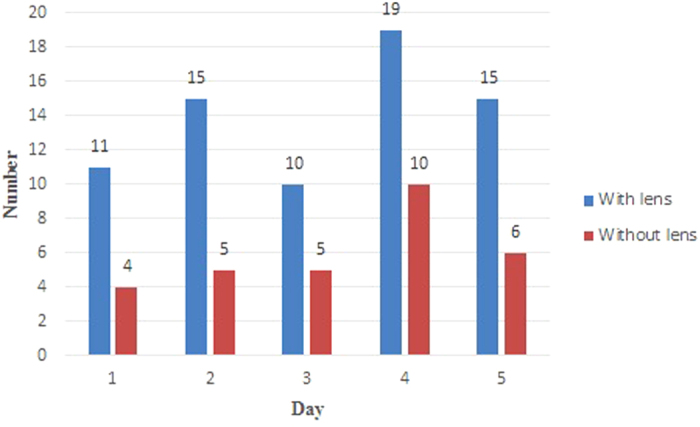
The number of caught mosquitoes due to the linear current driving LED with lens and that without lens for each day respectively.

In order to test the mosquito attraction capability, the LED light types were changed to attract mosquitoes using different light distribution patterns, the 3 W UV LED light bar shown in Fig. 1a and which is mounted by a lens array shown in Fig. 1c were used in the bug zapper respectively. The luminous intensity distribution measurements of the LED lights were conducted by using the ProMetric near field measurement system (PM-NFMS) developed by Radiant Vision Systems Co.. An optical power meter (1830-R) and an integrating sphere produced by Newport Co. were used to measure the LED lights output power. The LED lights without lens output power was 72.13 mw and the with lens LED light bars was 55.5 mw. Distribution curves of LED before and after lens deployment are shown in Fig. 1b and d, respectively. The results indicate that the beam divergence angle increased from 135 to 160 degrees by the lens installation, whereas the output light power decreased. In addition, the tests of bug zappers with different LED used outdoors in the same area and at the same time showed that the mosquito attraction effect of the wide-angle LED lights increased by 2.33 times (Fig. 2), which raw data are shown in Table 1. Thus, it can be certain that an increase in LED beam divergence angle can help attract mosquitoes, even the output power of the LED light is reduced due to the lens.

Apart from LED beam divergence angle, using pulse signals to drive the LED was also found to give help in mosquitoes catching. In order to explore the effect, the LED light bar without lens was pulse-driven in two bug zappers that only differed in the LED driving mode. One bug zapper named the experiment trapper, PWM signals were used to drive the LED light, whereas another bug zapper acted as the reference trapper a constant current driving mode was involved. In the reference trapper, it was with 0.128 amperes constant driving current and 1.5 W power consumption with the UV LED. For the experiment trapper, the driving frequency was changed in each test for investigating how the PWM frequency influenced on the insect attraction effect.

**Figure 3:**
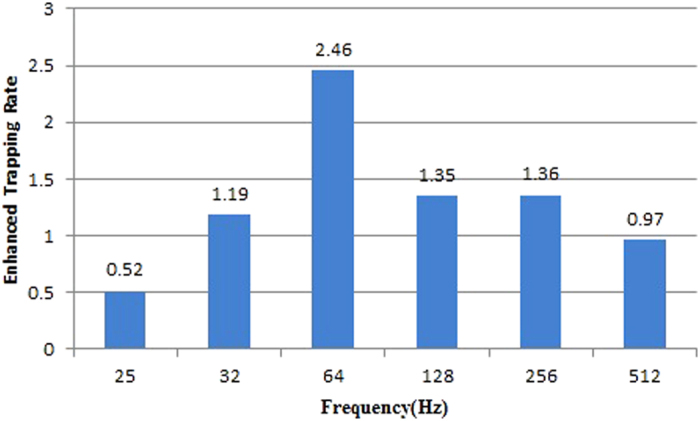
The enhanced trapping rates of using the no-lens UV LED light with different PWM driving frequency.

During the experiment, the two bug zappers were placed one meter from each other and were set from Mar. 22 to Jul. 17 in open spaces on our campus. They were turned on every day from 4 p.m. to 8 a.m. The LED was driven at PWM driving frequencies of 25 Hz, 32 Hz, 64 Hz, 128 Hz, 256 Hz, and 512 Hz respectively for experiments, and the LED duty cycle was controlled to maintain the average LED power consumption at 0.9 W for each test. Measurements at each frequency were conducted for five consecutive days. The number of trapped insects was recorded every day. The number in the experimental trapper relative to that in the reference trapper is defined as the enhanced trapping rate. According to the experimental results shown in Fig. 3, it was found that the enhanced trapping rate differed depending on the driving frequency, with the maximum enhanced trapping rate at 64 Hz and relatively low rate at frequencies lower than 32 Hz and higher than 256 Hz. At frequencies higher than 512 Hz, the attraction of insects was even worse than that of the reference trapper. These findings indicated that even when the power consumption of PWM-driven LEDs is 0.6 (0.919 W/1.536 W) times lower than that of the constant current driven LED, as shown in Table 2, PWM LEDs still can outperform the latter in the ability to trap mosquitoes. Particularly, at the driving frequency of 64 Hz, the trapped quantity of mosquitoes is 2.46 times increased.

**Figure 4:**
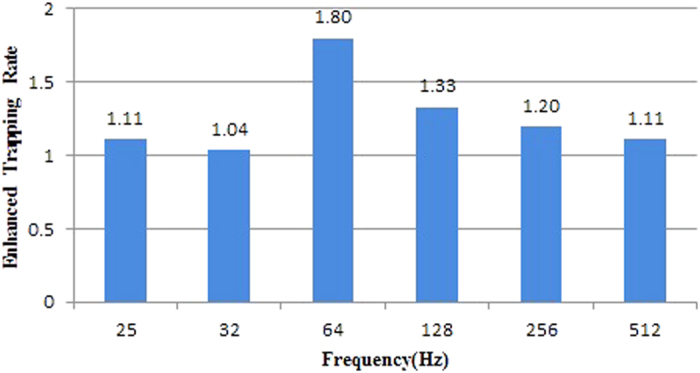
The enhanced trapping rate graph under the use of no-lens 1.5 W PWM LED.

Figure 4 shows the enhanced trapping rates at different driving frequencies under the conditions of higher power consumption of PWM-driven LED, which was increased from 0.9 W to 1.5 W, which is the same as the LED driven power of the reference trapper. The results showed that when the power consumption of PWM LED increases, bug zapper trapping capability at 64 Hz decreases but remains the highest among other driving frequencies and the difference between driving frequencies in terms of the trapping capability becomes smaller. These findings means that when PMM LED power consumption is increased to 1.5 W, LED duty cycle should be increased 1.8 times, which may result in longer LED conduction time and reduced light contrast ratio, so making the efficiency of the PWM driving mode and the linear current driving mode similar. Thus, it can be inferred that the effect of contrast ratio on the attraction of insects is higher than that of light output power.

**Figure 5:**
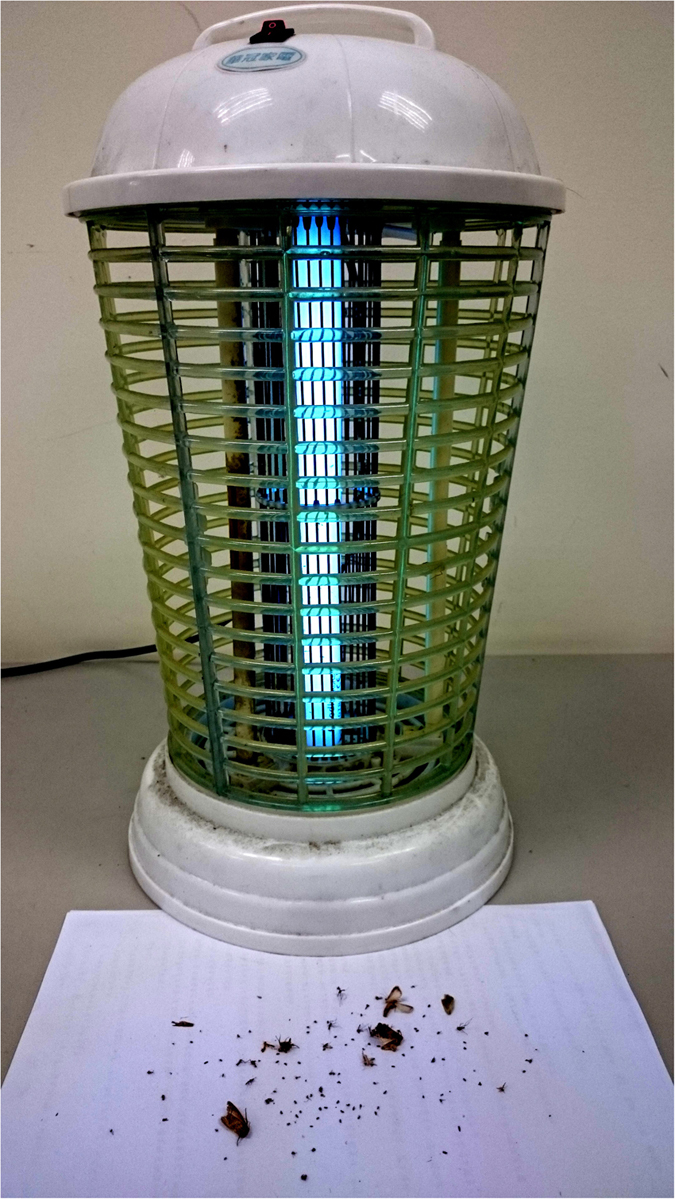
The fluorescent lamp bug zapper used in experiments of the study.

**Figure 6:**
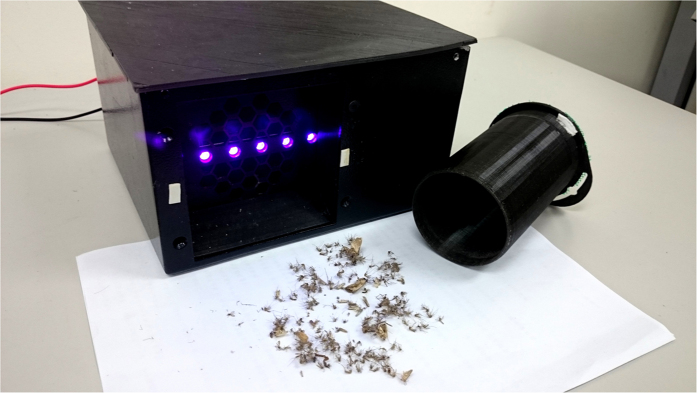
The UV LED bug zapper constructed for experiments of the study.

**Figure 7:**
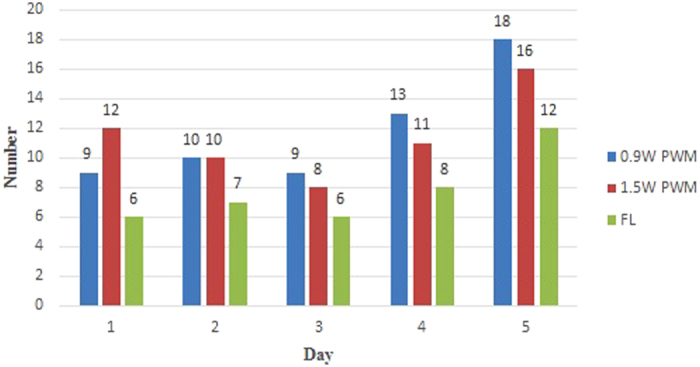
The graph of the number of trapped mosquito by the bug zappers with FL lamp, 0.9 W PWM LED and 1.5 W PWM LED respectively.

Finally, this study compared a 10 W FL bug zapper (Fig. 5) and two LED bug zappers (Fig. 6) equipped with 0.9 W PWM-UV LEDs (64 Hz) and 1.5 W PWM-UV LEDs (64 Hz), respectively. Measurements were conducted 24 hours for five days. The experiment results are presented in Fig. 7 and Table 1. The results showed that the number of trapped mosquitoes in the bug zapper using 0.9 W PWM LED was 1.04 times higher than that using 1.5 W PWM LED. And the number of trapped mosquitoes in the 1.5 W PWM LED was 1.46 times higher than in the FL bug zapper (Table 1). The power consumption of the 1.5 W UV LED light was only 15% (1.5/10 × 100% = 1.5%) of the 10 W FL lamp’s.

## Discussion and Conclusions

The experimental results in this study show that the light output angle and flicker frequency of 395 nm UV LED can greatly improve bug zapper capability to attract mosquitoes. And, even under lower input electrical power, flickering light still can have a better attraction effect than the linear current driving one. Conclusively, adjustment of LED light output angles and the use of PWM signals can reduce bug zapper power consumption and increase the capability of trapping mosquitoes.

### Research Principles

Due to low UV emission efficiency of FL light sources, most studies addressing the issue of bug zapper efficiency have focused on how to improve power consumption, for example, making use of LED as the insects attracting light source. However, despite higher efficiency and lower power consumption of LED as compared with FL, traditional linear LED driving method still consume too much electric power for a bug zapper used outdoors or outside the area of utility power supply. This study investigates the use of LED with pulse-width modulated signals and LED light energy distribution with lens control to improve a bug zapper’s mosquito eradication ability and reduce its power consumption. This can increase the operating time of batteries responsible for energy storage, as well as their service life due to frequent charge/discharge cycles.

Signals with high contrast ratio can make the light source location more obvious and attract living things. In an experiment conducted by David *et al*.^11^, proposes a flickering light source showed that critical flicker frequency (CFF) of participants’ measured brain responses ranged between 25 and 50 HZ. They also find that the responses of human vision vary with the modulation frequency of optical signals distinctly. If the increased contrast ratio can enhance biological vision, it is expected that the mosquito collection ability of bug zappers could also be improved by PWM optical signals. If the attraction of mosquitoes to the light source rises, mosquito eradication efficiency and the requirement for LED driven power can thus be relieved.

**Figure 8:**
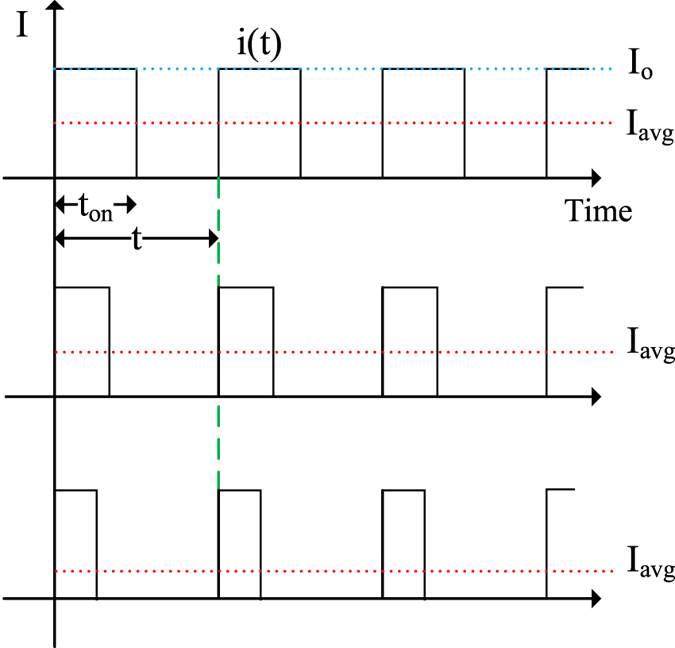
Pulse width modulated driving current of the UV LED for bug zapper in the study.

In PWM techniques, consumed power of LED is adjusted by changing the duty cycle of on-time and off-time electronic elements from 0% to 100% to control the average current *I*_*avg*_ (Fig. 8). The average current *I*_*avg*_of a PWM-LED circuit is calculated as in Equation (1),


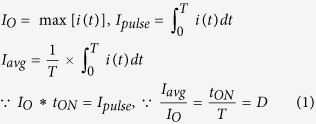


where i(t) is the instantaneous value of the current signal, *I*_*o*_ is the peak forward current of LED during switch conduction time, *I*_*pulse*_ is a unit pulse current*, I*_*avg*_ is the averaged LED current, t_ON_ is the switch conduction time, and D is the duty cycle. As seen from Equation (1), the average LED current *I*_*avg*_ and duty cycle D is positively correlated.

**Figure 9:**
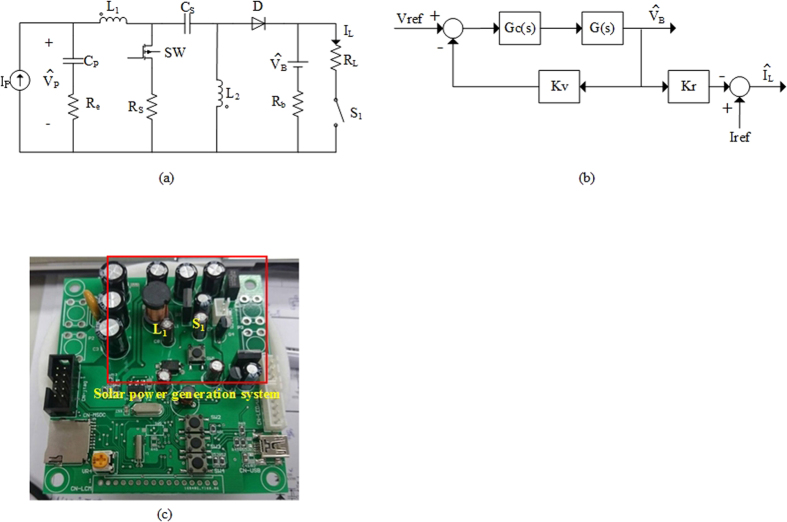
(**a**) The model of solar power generation system used in the study. (**b**) The controller of the solar power generation system used in the study. (**c**) The prototype of the power management circuit we design and produce for the study.

In order to control the power consumption of UV LED precisely, the equations are used for equalizing operating behavior of the solar power supply system. The system circuit is first converted into the equivalent circuit (Fig. 9a), and which is then solved using the Laplace transform to accomplish the transfer function as shown in Equation (2)^12^. To ensure accuracy and stability of the system, the transfer function of a proportional–integral–derivative controller (PID controller) needs to be added as in Equation (3). The transfer function of a complete system and a PID controller form a comprehensive control core (Fig. 9b). After being stylized, the control core equation is entered into the micro control unit (MCU) to obtain a digital control core. During system operations, the MCU can use the digital controller to calculate output voltage V_B_ and current I_L_ of the detection system and convert them into duty cycles of SW and S_l_ transfer switches to ensure pulse signal generation and pulse-width modulation.


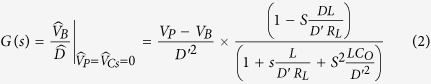


G(s) is the transfer function of a photovoltaic (PV) charge system; D is a duty cycle; D′ is 1-D; C_O_ is a battery seen as the equivalent output filter capacitor^12^.


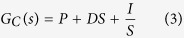


G_C_(s) is the system controller; P is the proportional controller; I is the integral controller; D is the derivative controller.

### Experimental setups

**Figure 10:**
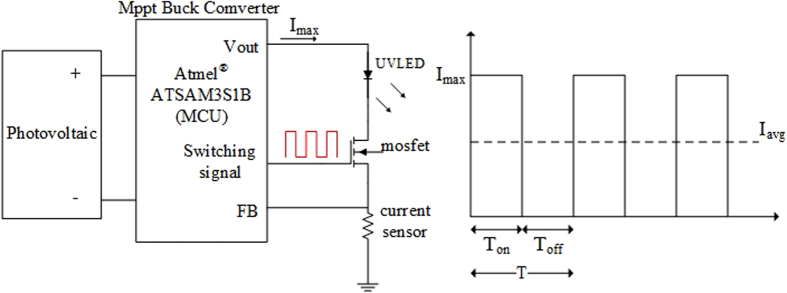
The PWM signals generator circuit for LED used in the study.

In addition to verify that a PWM UV-LED module can reduce power consumption and enhance the function of mosquito attraction, the experiments in this study explored the effect of light modulation frequency and light field distribution on the attraction of mosquitoes. Due to a positive relation between LED radiance and LED driven current, a PWM driving signal can modulate LED output through the control over the duty cycle of current signals. Figure 10 illustrates the electrical circuit generating PWM current signals.

**Figure 11:**
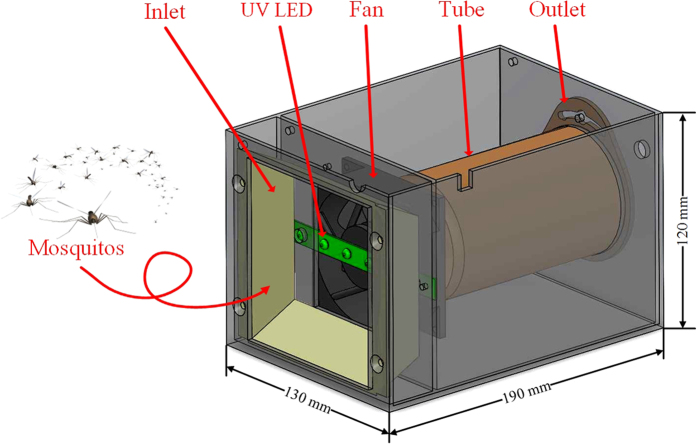
The opto-mechanical structure of the bug zapper constructed for the experiments.

In order to investigate attraction of mosquitoes, a bug zapper system shown in Fig. 11 is established by integrating an opto-mechanical system and an electrical power module. The structure includes a plastic box with an air inlet in front, a set of 395 nm 3 W UV LED light 3 mm after the inlet (Currently commercial UV LEDs typically have the wavelength of 395 nm, and owns more efficiency than the UV light with shorter wavelength, such as 365 nm.), and a 12 VDC/0.07 A/80 mm square direct current (DC) fan 10 mm from the inlet. On the rear of the fan, there was a length of 75 mm diameter tubular mosquito collection box. The bug zapper attracted mosquitoes by UV rays emitted by the LED light source, making the insects approach the suction inlet and forcing them into the collection tube due to the effect of wind suction generated by the DC fan. Mosquitoes are trapped in the collection box with the DC fan continuously operating, resulting in the insects’ demise due to air dry or starvation. The power module consisted of a 7 W thin-film solar cell and a power management circuit. Light energy collected by the solar cell was converted and stored in a 12 V_DC_/7.2 AH lead-acid battery inside the system to supply necessary electric power for UV LED and fan operations according to the system settings.

## Additional Information

**How to cite this article**: Liu, Y.-N. *et al*. Enhancement of mosquito trapping efficiency by using pulse width modulated light emitting diodes. *Sci. Rep.* **7**, 40074; doi: 10.1038/srep40074 (2017).

**Publisher's note:** Springer Nature remains neutral with regard to jurisdictional claims in published maps and institutional affiliations.

## Figures and Tables

**Table 1:** The count data of trapped insects by the bug zappers.

Date (2016)	Parameter	Mosquito			Non- Mosquito	
		CQ	CA/Aeg/Alb	Total	Moths	Others
Mar.22–21	PWM-25 Hz-0.9 W	14	CA x1	15	20	64
	Linear-1.5 W	29	0	29	31	82
		enhanced trapping rate =(15/29 = 0.52)				
Mar.31-Apr.4	PWM-32 Hz-0.9 W	18	CA x1	19	18	185
	Linear-1.5 W	16	0	16	26	167
		enhanced trapping rate =(19/16 = 1.19)				
Apr.18–22	PWM-64 Hz-0.9 W	52	CA x2	54	17	143
	Linear-1.5 W	19	CA x3	22	15	136
		enhanced trapping rate =(54/22 = 2.46)				
Apr.27-May.1	PWM-128 Hz-0.9 W	41	CA x3, Aeg x2	46	18	145
	Linear-1.5 W	31	Aeg x3	34	12	118
		enhanced trapping rate (46/34 = 1.35)				
May.5–9	PWM-256 Hz-0.9 W	30	CA x3, Aeg x1	34	19	164
	Linear-1.5 W	24	CA x1	25	14	203
		enhanced trapping rate =(34/25 = 1.36)				
May.10–14	PWM-512 Hz-0.9 W	32	CA x2	34	31	154
	Linear-1.5 W	31	CA x3, Alb x1	35	20	185
		enhanced trapping rate =(34/35 = 0.97)				
May.15–19	PWM-25 Hz-1.5 W	26	CA x2, Aeg x2	30	27	86
	Linear-1.5 W	23	CA x4	27	25	103
		enhanced trapping rate =(30/27 = 1.11)				
May.20–24	PWM-32 Hz-1.5 W	20	CA x2	22	41	89
	Linear-1.5 W	21	0	21	33	81
		enhanced trapping rate =(22/21 = 1.04)				
May.27–31	PWM-64 Hz-1.5 W	24	CA x3	27	16	82
	Linear-1.5 W	15	0	15	20	79
		enhanced trapping rate =(27/15 = 1.8)				
Jun.1–5	PWM-128 Hz-1.5 W	15	CA x1	16	30	61
	Linear-1.5 W	11	CA x1	12	36	64
		enhanced trapping rate =(16/12 = 1.33)				
Jun.15–19	PWM-256 Hz-1.5 W	12	0	12	32	74
	Linear-1.5 W	10	0	10	24	73
		enhanced trapping rate =(12/10 = 1.2)				
Jun.20–24	PWM-512 Hz-1.5 W	20	0	20	25	79
	Linear-1.5 W	17	CA x1	18	21	73
		enhanced trapping rate =(20/18 = 1.11)				
Jul.13–17	With Lens- Linear-1.5 W	69	Alb x1	70	21	62
	Without Lens- Linear-1.5 W	29	CA x1	30	19	80
		the mosquito attraction effect (70/30 = 2.33)				
Qct.29-Nov2	PWM-64 Hz-0.9 W	51	CA x5, Alb x3	59	29	139
	PWM-64 Hz-1.5 W	52	CA x4, Alb x1	57	38	146
	FL-10 W	31	CA x8	39	186	281
PWM LED and FL power consumption rate (1.5/10 × 100% = 15%)				PWM LED trapping rate (59/57 = 1.04)		

Abbreviation: CQ: Culex Quinquefasciatus; CA: Culex Annulus; Aeg: Aedes Aegypti;Alb: Aedes Albopictus.

Non- Mosquito others: psychodidae, muscidae, etc.

**Table 2:** The list of power consumption.

	measured power consumption		Total (DC Fan + UV LED)
	DC Fan	UV LED	
PWM-0.9 W	0.84 W	0.919 W	1.759 W
Linear-1.5 W	0.84 W	1.536 W	2.376 W
LED power consumption rate (0.919/1.536 = 0.598)			
Total power consumption rate (1.759/2.376 = 0.740)			
